# Nucleation of mercury sulfide by dealkylation

**DOI:** 10.1038/srep39359

**Published:** 2016-12-19

**Authors:** Mironel Enescu, Kathryn L. Nagy, Alain Manceau

**Affiliations:** 1 Laboratoire Chrono Environnement, Université de Franche-Comté, CNRS, 25030 Besançon, France; 2 Department of Earth and Environmental Sciences, MC-186, 845 West Taylor Street, University of Illinois at Chicago, Chicago, Illinois 60607, United States; 3 ISTerre, Université Grenoble Alpes, CNRS, CS 40700, 38058 Grenoble, France

## Abstract

Metal sulfide minerals are assumed to form naturally at ambient conditions via reaction of a metallic element with (poly)sulfide ions, usually produced by microbes in oxygen-depleted environments. Recently, the formation of mercury sulfide (β-HgS) directly from linear Hg(II)-thiolate complexes (Hg(SR)_2_) in natural organic matter and in cysteine solutions was demonstrated under aerated conditions. Here, a detailed description of this non-sulfidic reaction is provided by computations at a high level of molecular-orbital theory. The HgS stoichiometry is obtained through the cleavage of the S-C bond in one thiolate, transfer of the resulting alkyl group (R’) to another thiolate, and subsequent elimination of a sulfur atom from the second thiolate as a thioether (RSR’). Repetition of this mechanism leads to the formation of RS-(HgS)_n_-R chains which may self-assemble in parallel arrays to form cinnabar (α-HgS), or more commonly, quickly condense to four-coordinate metacinnabar (β-HgS). The mechanistic pathway is thermodynamically favorable and its predicted kinetics agrees with experiment. The results provide robust theoretical support for the abiotic natural formation of nanoparticulate HgS under oxic conditions and in the absence of a catalyst, and suggest a new route for the (bio)synthesis of HgS nanoparticles with improved technological properties.

Nucleation of metal sulfide solids typically occurs when solubility is exceeded by elevated concentration of reduced sulfur, metal cation, or both components^1^,^2^. In environmental aquatic systems, metal ions are commonly complexed with natural organic matter or inorganic anions, including sulfide, and free sulfide ions (S(-II)) produced mainly by dissimilatory sulfate reducing microbes^3^,^4^ are considered necessary for solid nucleation. Sulfide can also be generated in the laboratory from intracellular cysteine by photosynthetic aerobic microorganisms^5^,^6^ and from decomposition of sulfur compounds, such as thioglycolic acid, thioglycerol, dithiocarbamate, thioacetamide, and cystine, by hydrothermal, solvothermal, and biomimetic synthesis routes, sonochemical reaction, microwave irradiation, and hydrolysis^7^,^8^,^9^,^10^,^11^,^12^,^13^,^14^,^15^,^16^,^17^,^18^.

Recently it was shown that sulfide ions were not required to form a metal sulfide solid^19^. Metacinnabar (β-HgS) precipitated directly from linear Hg-thiolate complexes (Hg(SR)_2_) in natural organic matter (NOM) and from Hg-dicysteinate complexes (Hg(Cys)_2_) in aerated and deaerated aqueous solutions in the dark without a catalyzing agent. These results are relevant to soil and aquatic systems, especially in cases where organo-sulfide is the dominant sulfide source. The reaction was rather slow and took several days for Hg(II) complexed to NOM at a concentration of 30–200 mg of Hg/kg of NOM dry weight (ppm). A global reaction pathway was proposed that has similarities to one suggested for β-HgS precipitation in sodium hydrosulfide (NaHS) solution^20^,^21^. In its reaction with NaHS, Hg(II) initially forms an unstable low coordination chain-type complex (–S-Hg-S-Hg-S-) that rapidly transforms to a four-coordinate mercury sulfide with the short range ordering of β-HgS. The disordered β-HgS nanostructures eventually yield β-HgS crystals. In the case of thiolate as the source of reduced sulfur, the starting reactant is the linear Hg(SR)_2_ complex (RS-Hg-SR), which is the most stable coordination of mononuclear Hg with thiolate ligands at neutral and acidic pH^22^,^23^. Because β-HgS nanostructures appear rapidly once –S-Hg-S-Hg-S- chains are formed in sulfidic solution^20^, we infer that formation of the chain structure limits the rate of formation of β-HgS from Hg(SR)_2_. The pathway proposed^19^ for chain formation in natural organic matter is the cleavage of the S-R bond according to the reaction:


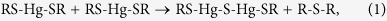


followed by growth of the chain through the addition of new Hg(SR)_2_ complexes:


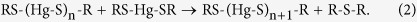


Given that β-HgS also was obtained from Hg-dicysteinate complexes (Hg(Cys)_2_)^19^, the R group in natural organic matter can be an alkyl ligand of the general form CH_2_-R’. Thus, reaction (1) involves the cleavage of a S-C bond in an R’-CH_2_-S-Hg-S-CH_2_-R’ entity. It can be described as a transfer of an alkyl group between two aliphatic thiolates (SR^−^) followed by dissociation of the resultant R-S-R thioether and bonding of Hg to the exposed S. Elimination of one sulfur from two Hg(SR)_2_ complexes decreases the S to Hg ratio from 4:2 to 3:2 in the mercury product and triggers the formation of HgS when the reaction is repeated as in (2).

Here, we present quantum chemical calculations of the structure and energetics of the transition state in reaction (1) that support our previous experimental results. The results show that the proposed dealkylation of the Hg(SR)_2_ complex is thermodynamically allowed and has an activation free-energy barrier consistent with the kinetics of formation of β-HgS in natural organic matter. We also discuss how cinnabar (α-HgS) and metacinnabar are formed by the proposed reaction mechanism directly from Hg-thiolate complexes in the absence of any catalyst or external reagent.

## Results

### Structural mechanism of dealkylation

**Figure 1:**
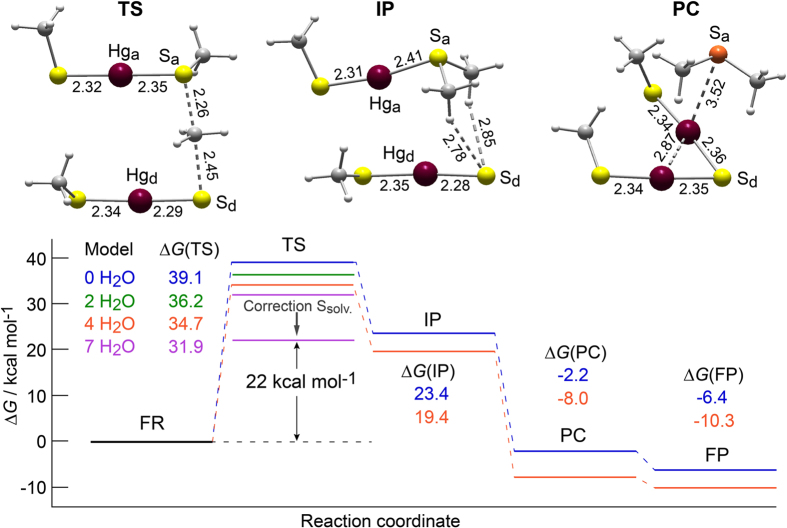
Mechanistic pathway of formation of a Hg(II) sulfide dimer by dealkylation^38^ of two Hg-thiolate complexes. Gibbs free energy diagram (at 298 K and 1 atm) of the cleavage of the S-C bond by an alkyl group transfer between two linear Hg-thiolate complexes, and optimized structures for the reaction pathway with four explicit water molecules (not shown for clarity). The height of the activation-energy barrier for the alkyl group transfer relative to the free reactant state decreases from 39.1 kcal mol^−1^ to 36.2 kcal mol^−1^ with two explicit water molecules, to 34.7 kcal mol^−1^ with four, and to 31.9 kcal mol^−1^ with seven. The final value, corrected for overestimation of the solvation entropy in the continuum solvation models is 22 kcal mol^−1^. The same correction applies to the IP and PC states (corrected levels not shown). FR = free reactants; TS = transition state; IP = intermediate product; PC = product complex; FP = free products. Bond lengths are in angstroms. Dark red, Hg; yellow, thiolate sulfur SR^−^ and sulfide sulfur HgSHg; orange, thioether sulfur RSR; dark gray, C; light gray, H. Cartesian coordinates are given in the Supplementary Materials.

According to (1), the free reactants (FRs) are two linear Hg-thiolate complexes of formula RS-Hg-SR. The R group was represented in the computational work by a methyl group (CH_3_). This simplification has been validated previously on stability calculations of Hg(II) complexes with thiolate and thioether ligands^22^,^24^, and is also justified by the independence of the dissociation energy of the R−SH bond with respect to the nature of the R radical^25^. The direct transfer of an alkyl group between the two Hg-thiolate complexes may be regarded as a nucleophilic substitution with two sulfur atoms as nucleophilic centers. Thus, the three directly interacting atoms, that is, the donor sulfur (S_d_), the acceptor sulfur (S_a_), and the C atom of the CH_3_ group, should be collinear in the transition state (TS) to provide an adequate overlap of orbitals (Fig. 1). Based on the equivalence of the four S atoms, the transition state has a configurational degeneracy of eight. It decays to an intermediate product (IP) in which the S_d_ atom is placed nearly equidistant with respect to the two CH_3_ groups carried by S_a_. An internal rearrangement of the system leads to a more stable configuration for the product complex (PC), in which the S_d_ atom bonds to the Hg atom of the acceptor complex (Hg_a_) to form the mercury sulfide dimer Hg_2_S_3_(CH_3_)_2_. The scan of the potential energy surface with respect to the S_d_-Hg_a_ distance shows that this rearrangement occurs with practically no energy barrier (Supplementary Fig. S1). Simultaneously, the CH_3_-S_a_-CH_3_ group (thiodimethane) moves away to a Hg_a_-S_a_ distance of 3.52 Å. It can further dissociate from the mercury sulfide dimer, leaving the two as free products (FPs).

### Energetics of dealkylation

The transition state has an activation free-energy barrier of 39.1 kcal mol^−1^ without water molecules in the reaction core. Better estimates of the free energy are obtained when explicit water molecules are added to Hg(II) complexes to account for strong short-range hydrogen bonding interactions between the anion (here CH_3_S^−^) and the solvent^26^. The length of the S_d_…H hydrogen bonds effectively decreased from 2.37 Å in the free reactants to 2.24 Å in the transition-state structure when two water molecules were placed near the S_d_ atom, thus confirming the importance of solute-solvent covalent interactions^26^,^27^ (see Supplementary Materials). Overall, the activation energy decreased to 36.2 kcal mol^−1^ with two explicit water molecules, 34.7 kcal mol^−1^ with four, and 31.9 kcal mol^−1^ with seven (Fig. 1 and Supplementary Fig. S2). In the model with seven water molecules, the specific interactions between the three reactive ligands, S_a_, S_d_. and CH_3_, and the solvent are integrally taken into account since all the related hydrogen bonds are formed.

The energy barrier of 31.9 kcal mol^−1^ is lowered to about 22 kcal mol^−1^ after correcting for improper evaluation of the solvation entropy in the continuum solvent models^28^,^29^,^30^,^31^ (see Supplementary Materials). The same Gibbs free energy correction applies to the intermediate product (IP) and to the product complex (PC). To compare with experiment, the range of reaction times reported for the formation of β-HgS in natural organic matter and from Hg(Cys)_2_^19^ indicates an energy barrier on the order of 24 kcal mol^−1^, as estimated from Eyring’s formula^32^ for the reaction rate constant. The predicted value is close enough to the experimental value to validate the proposed reaction mechanism.

One might expect the transfer of a methyl group between two identical atoms (S_d_ and S_a_) to be reversible. The back transfer of the methyl group here is unlikely because of the internal rearrangement of the system leading to the product-complex state. This state is more stable than the free-reactant state by −8.0 kcal mol^−1^, and further decays to the free product state which is −10.3 kcal mol^−1^ lower in energy than the free reactants (as calculated with 4 H_2_O, Fig. 1). Although the Hg atoms are not directly involved in the nucleophilic substitution, they play a key role in the product rearrangement through the intermolecular Hg-S forces and the attractive intramolecular short-range Hg-Hg interactions of van der Waals type^33^,^34^,^35^.

An alternative to the dealkylation reaction is the insertion of the Hg atom from one linear Hg-thiolate complex between the S and C atoms of the S-C bond from the other complex, as observed in coordination complexes with Co(III) and W(III)^36^,^37^. Calculations performed for two possible reaction pathways each gives a high Gibbs free energy for the intermediate product (see Supplementary Materials).

### Formation of HgS

**Figure 2:**
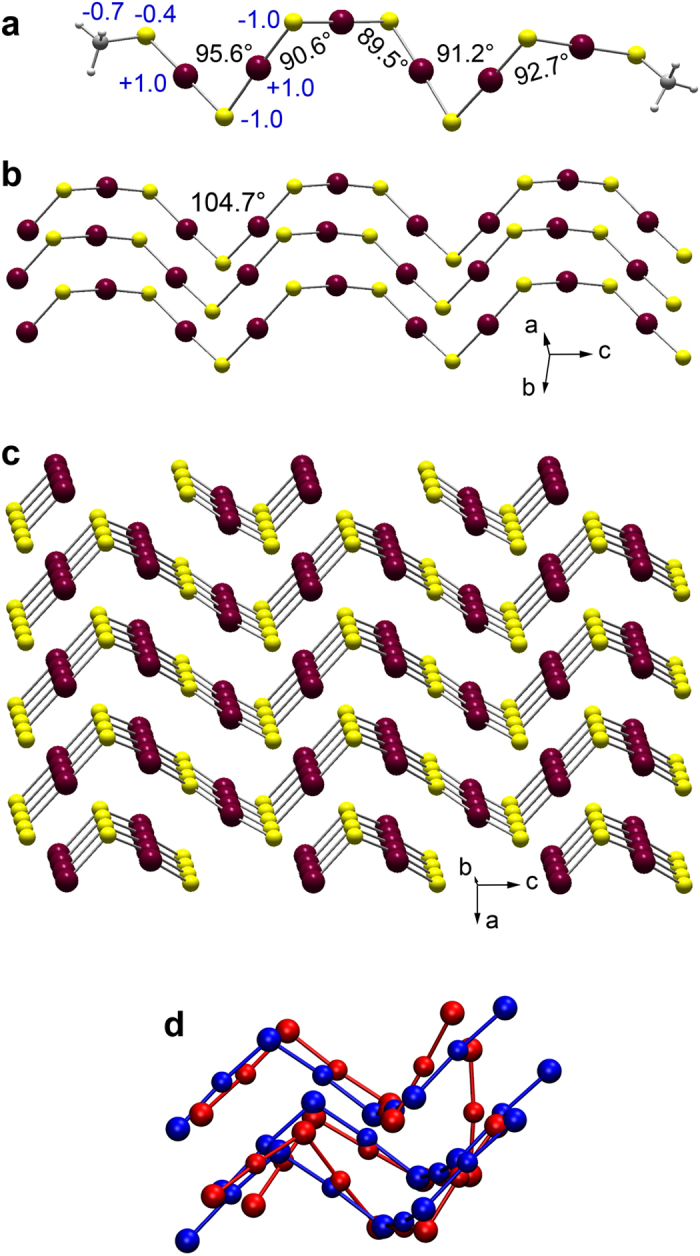
Formation of cinnabar by association of -S-(Hg-S)_n_-Hg-S- chains. (**a**) Hg_6_S_7_(CH_3_)_2_ model optimized in aqueous solution with the CPCM model. Hg-S-Hg angles are in black. Atomic charges, in units of elementary charge e and calculated by natural population analysis (NPA)^61^, are in blue. Hg (dark red) has a natural charge of +1.0 e, sulfide S (yellow) of -1.0 e, thiol S (yellow) of −0.4 e, C (dark gray) of ^−^0.7 e, and H (light gray) of +0.2 e (not represented). (**b**) Three parallel -S-(Hg-S)_n_-Hg-S- chains in cinnabar^38^. (**c**) Cinnabar as an assemblage of replicated chains. d) Best superposition of the trimer 3[Hg_4_S_5_H_2_] optimized in aqueous solution (red, H atoms not shown) and three fragments of adjacent Hg_4_S_5_ chains from the structure of cinnabar (blue).

The -S-(Hg-S)_n_-Hg-S- chain formed by repetition of the dealkylation mechanism has a specific conformation (Fig. 2a). Because the sulfur ligands are linearly coordinated to Hg, the chain conformation is completely determined by the Hg-S-Hg angle and the S-S-S-S dihedral angle defined by four successive S atoms. The first angle ranges from 89.5° to 95.6° and the second from −86.7 to −101.4° in the optimized Hg_6_S_7_(CH_3_)_2_ model shown in Figure 2a. This conformation is close to that in cinnabar (α-HgS), which has infinite chains throughout its structure with a Hg-S-Hg angle of 104.7° and a dihedral S-S-S-S angle of −98.5°^38^ (Fig. 2b,c). A primitive α-HgS nanostructure, as observed experimentally in aqueous solution with sodium hydrosulfide (NaHS) before the subsequent formation of β-HgS^20^, is obtained by optimizing the geometry of three HS-(Hg-S)_3_-Hg-SH chains in aqueous solution (Fig. 2d). Once formed, the zigzag -S-(Hg-S)_n_-Hg-S- chains self-assemble to make the trimer 3[Hg_4_S_5_H_2_], which is geometrically comparable to three neighboring Hg_4_S_5_ units in cinnabar. The coordination around the Hg atoms in α-HgS is “2 + 4”, with two short intra-chain Hg-S bonds 2.37 Å in length and four long inter-chain Hg-S bonds of 3.10–3.29 Å^38^. Similarly, the cohesion of the HS-(Hg-S)_3_-Hg-SH aggregate is realized by inter-chain Hg-S bonds ranging from 3.15 Å to 3.37 Å. In aqueous solution with sodium hydrosulfide (NaHS), the early-formed 2 + *n (n* < 4) coordination is unstable and quickly evolves to a 4 coordination (i.e., tetrahedral) with the local ordering of metacinnabar (β-HgS)^20^. The same transformation is assumed to occur in natural organic matter because only nanoparticulate β-HgS is detected^19^. The 2 + *n* to 4 transition is however difficult to model because β-HgS is thermodynamically metastable at room temperature^21^,^39^,^40^.

We have proposed a new mechanism for cleavage of the S-C bond of thiolate in the presence of Hg(II), based on the transfer of one alkyl group (R) between two linear Hg-thiolate complexes (Hg(SR)_2_), and elimination of a sulfur atom by formation of a thioether (RSR). This reaction initially produces a mercury sulfide dimer and subsequently mercury sulfide clusters if replicated. The mechanism provides robust theoretical support for the experimental nucleation of nanoparticulate metacinnabar from Hg(II)-thiolate complexes in natural organic matter and from Hg-dicysteinate complexes^19^. It also offers an explanation for the occurrence of metacinnabar under oxic conditions in soils^19^,^41^,^42^, for what has been termed ‘old’ soil mercury, i.e., mercury deposited from the atmosphere that becomes relatively recalcitrant within weeks to months^43^,^44^,^45^, and for metal sulfides associated with dissolved natural organic matter in river water^46^. The nucleation of HgS particles from Hg-thiolate complexes is significantly slower, therefore yields less defective structures than with free sulfides^20^ because the sulfur release is controlled by a non-negligible energy barrier. This could lead to interesting effects on the size, shape, and crystallinity of metacinnabar nanocrystals and improved control over (bio)synthesis, structures, properties, and functionality of this technologically important material^10^,^13^,^15^,^18^.

## Method

Calculations were performed with GAUSSIAN 09^47^ using a computational method validated previously on Hg-thiolate complexes^22^. All calculations were performed in aqueous solutions using the supermolecule-continuum solvent model, as developed in the framework of the conductor-like polarizable continuum model CPCM^48^, which allows explicit water molecules in contact with the reactants in a continuum bulk solvent. The geometry optimizations were performed using the second-order Møller-Plesset perturbation theory (MP2)^49^, and single point energies were evaluated using the hybrid method Integrated Molecular Orbital and Molecular Orbital (IMOMO)^50^, ONIOM version^51^,^52^. The IMOMO method combines calculations of energies at two levels of theory: a higher one applied to a limited part of the system (called the “model system”, here the Hg-thiolate complexes without explicit water molecules) and a lower one applied to the whole system (called the “real system”) which includes water molecules. The model system was treated at the coupled cluster level of theory with single and double substitutions and corrections for triple substitutions (CCSD(T))^53^,^54^,^55^,^56^ and the real system was treated at the MP2 level. The C, H, and O centers were treated using the aug-cc-pVDZ basis set^57^ while the S centers were represented at the aug-cc-pVTZ level^58^. The mercury atom was treated using the Stuttgart-Dresden-Bonn quasirelativistic pseudopotentials (SDD)^59^ for the core electrons and the associated valence basis set (describing 20 valence electrons of Hg). Two polarization functions of *f* type taken from ref.^60^ were added in order to ameliorate the Hg basis set. Other computational details are given in the Supplementary Materials.

## Additional Information

**How to cite this article:** Enescu, M. *et al*. Nucleation of mercury sulfide by dealkylation. *Sci. Rep.* **6**, 39359; doi: 10.1038/srep39359 (2016).

**Publisher's note:** Springer Nature remains neutral with regard to jurisdictional claims in published maps and institutional affiliations.

## Supplementary Material

Supplementary Information
