# Supplementary material for: Nucleation of mercury sulfide by dealkylation
Source: Sci Rep. 2016 Dec 19;6:39359. doi: 10.1038/srep39359 (PMC5171843; doi:10.1038/srep39359)
Supplement: Supplementary Information [file srep39359-s1.pdf]

Supplementary Information

# Nucleation of mercury sulfide by dealkylation

Mironel Enescu, Kathryn L. Nagy & Alain Manceau

**1. Evaluation of the Gibbs free energy**
**2. Energy profile for the Intermediate Product to Product Complex structural rearrangement**
**3. Effect of explicit water molecules on the dealkylation energy barrier**
**4. Correction of the contribution of translation-rotation solvation entropy to the energy barrier**
**5. Cleavage of the S-C bond by direct insertion of a Hg atom**
**6. References**
**7. Gaussian 09 input files and optimized Cartesian coordinates**

## 1. Evaluation of the Gibbs free energy

Stationary points of the reaction pathway were identified by scanning the potential energy surface (PES) against the location of all atoms in the system without water molecules. The Gibbs free energy in aqueous solution ($G_{aq}$) for the molecular state (M) at each stationary point was evaluated according to:

$$G_{aq}(M) = E_{CCSD(T)}(M) + \Delta G_{corr}(M), \qquad (1)$$

where $E_{CCSD(T)}(M)$ is the total electronic energy (i.e., potential energy) including the solvent effects, and $\Delta G_{corr}(M)$ is the thermal and entropic contributions to $G$ calculated at 298 K and 1 atm. The thermal and entropic contributions were obtained by performing frequency calculations. The $G_{aq}(M)$ values were corrected for the difference between the standard molar concentrations in solution and gas phase. Since the CPCM solvation energy does not include the energy of formation of solvent cavities, we evaluated this contribution separately for the final structures using the SMD version[1] of the polarizable continuum dielectric model (PCM). The correction for this effect on the calculated energy barriers was small, in the range of 1-2 kcal mol$^{-1}$. The conformational degeneracy of the transition state was taken into account by adding to $G$ the term $-RT*ln(g)$, where $g$ is the degeneracy, $R$ the gas constant, and $T$ the absolute temperature.

Energetic values in the presence of water molecules were calculated as follows. The geometry of the stationary points were reoptimized with explicit water molecules added in contact to the main atoms involved in the chemical reaction. The whole system ("real system"[2]) was optimized at the MP2 level of theory, and the "model system"[2] including all atoms except those belonging to water molecules was treated at the CCSD(T) level of theory. The ONIOM energy[2,3] was calculated according to:

$$E_{ONIOM} = E_{CCSD(T)}(model) - E_{MP2}(model) + E_{MP2}(real). \qquad (2)$$

The three energy terms in eq. 2 were calculated in the presence of the bulk solvent (CPCM) and for the geometry optimized on the real system. Finally, the Gibbs free energy of the system was calculated from:

$$G_{aq} = E_{ONIOM} + \Delta G_{corr}(real) \qquad (1')$$

with $\Delta G_{corr}$(real) obtained by a frequency calculation performed at the MP2 level of theory on the optimized real system (i.e., in the presence of the bulk solvent).

The choice of the pure continuum solvation model has only a limited impact on the calculated Gibbs free energy. For instance, we previously obtained[4] $\Delta G$(FP) = -3.9 kcal mol$^{-1}$ for reaction (1) (main text) using the pure PCM model, compared to -6.4 kcal mol$^{-1}$ with the pure CPCM model in the present study (Fig. 1).

The convergence criteria used in the geometry optimizations were the default settings in the Gaussian 09 program :

| | |
|---|---|
| Maximum Force | 0.000450 |
| RMS Force | 0.000300 |
| Maximum Displacement | 0.001800 |
| RMS Displacement | 0.001200 |

**2. Energy profile for the IP to PC structural rearrangement**

The energy profile was obtained by constraining the $Hg_a$-$S_d$ distance to fixed values. The solvation model used here is the pure CPCM model (i.e., no explicit water molecules).

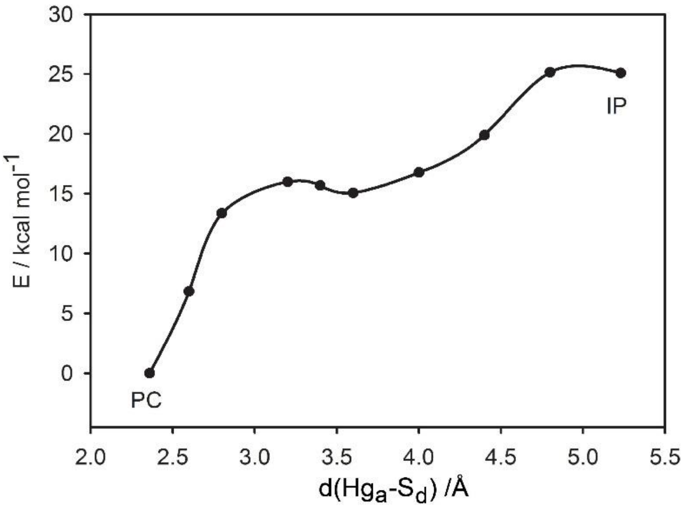


**Figure S1.** Energy profile for the rearrangement of the reaction product.

**3. Effect of explicit water molecules on the dealkylation energy barrier**

The transfer of the $CH_3^+$ group is associated with a significant reorganization of the electronic density around $S_d$, $S_a$, and the C atom of the transferred methyl group. This process is expected to be sensitive to the presence of solute-solvent hydrogen bonds because of their weak but definite covalent character[5]. Therefore, a more accurate evaluation of the reaction energy barrier is obtained when explicit water molecules are placed nearby the main atoms involved in the reaction[6]. This type of calculation was performed for 2, 4 and 7 explicit solvent molecules. Obviously, in all cases, the atoms from the solute around which the explicit water molecules were placed were the same in the free reactant (FR) and transition state (TS) structures (Table S1). In a first approach ($2H_2O$ model in Fig. 1), two water molecules were placed in contact with the $S_d$ atom of the two free reactants and of the TS complex. The length of the two $S_d$…H hydrogen bonds in the optimized structures decreased from 2.37 Å in the free reactants to 2.24 Å in the TS complex, thus proving the importance of solute-solvent covalent interactions. In a second approach ($4H_2O$ model in Fig. 1), two additional solvent molecules were placed in contact with the transferred $CH_3$ group because it is expected to become more electrophilic in the TS configuration. In a third approach ($7H_2O$ model in Fig. 1), the water molecules were placed as follows: three near Sd, two near Sa and two near the $CH_3$ group. This arrangement approximately corresponds to the first solvation shell of the directly

reacting atoms. In all model systems, the free reactant state is represented by two free supermolecules, each formed of a Hg(II)-methanethiolate complex ($CH_3S$-Hg-$SCH_3$) carrying several water molecules (0 and 2, 0 and 4, 2 and 5, respectively). The structure of the TS complex including the seven water molecules is shown in Fig. S2. Details of the reaction energetics are given in Tables S1-S3. $E$ is the total electronic energy, $H$ the enthalpy, $S_{tot}$ the total entropy, and $S_{tr}$ the translation-rotation entropy.

**Table S1**. Associations of the explicit water molecules and corresponding energy barriers at 298 K and 1 atm.

| Number of water molecules *n* | Position in the FR and TS structures | $\Delta E$ kcal mol$^{-1}$ | $\Delta H$ kcal mol$^{-1}$ | $\Delta G$ kcal mol$^{-1}$ |
|---|---|---|---|---|
| 0 | - | 29.9 | 31.1 | 39.1 |
| 2 | $S_d$(2) | 26.7 | 31.3 | 36.2 |
| 4 | $S_d$(2), $CH_3$(2) | 20.7 | 25.5 | 34.7 |
| 7 | $S_d$(3), $CH_3$(2), $S_a$(2) | 11.3 | 13.2 | 31.9 |

**Table S2**. Thermodynamic parameters (in kcal mol$^{-1}$) at 298 K and 1 atm for reaction (1) calculated using four explicit water molecules.

| Structures | $\Delta E$ | $\Delta H$ | $T\Delta S_{tot}$ | $T\Delta S_{tr}$ | $\Delta G$ |
|---|---|---|---|---|---|
| FR | 0 | 0 | 0 | 0 | 0 |
| TS | 20.7 | 25.5 | -14.6 | -19.5 | 34.7 |
| FP | -8.6 | -8.4 | 1.7 | -0.7 | -10.3 |

**Table S3**. Single point energies (in Hartree) for pure CPCM calculations ($E$(ccsd(t)) and for supermolecule calculations with seven water molecules ($E$(ONION)).

| | FR | TS | IP | PC | FP |
|---|---|---|---|---|---|
| *n* = 0 *E*(ccsd(t)) | -2056.35479 | -2056.307081 | -2056.345612 | -2056.382637 | −2056.37079 |
| *n* = 7 *E*(ONION) | −2590.290511 | −2590.272477 | - | - | - |

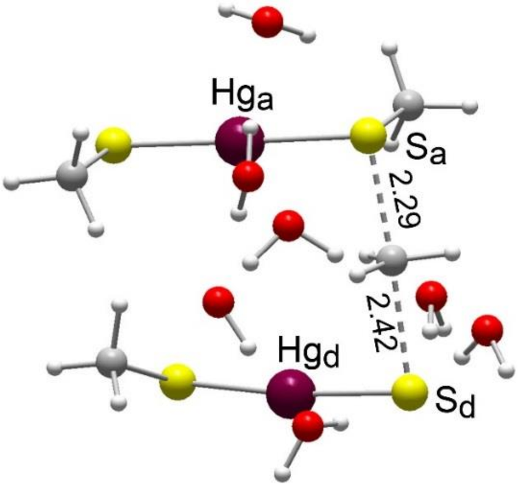


**Figure S2.** Structure of the TS configuration optimized with seven explicit water molecules

## 4. Correction of the contribution of translation-rotation solvation entropy to the energy barrier

A general source of error attributed to the continuum solvent models is the improper evaluation of the solvation entropy[7-10]. When the translation-rotation entropy of the molecule in solution is calculated using the same method as in the gas phase, this entropy is largely overestimated. The result is a systematic error in the evaluation of the Gibbs free energy difference between molecular states involving a different number of molecules. For instance, this error occurs systematically in the calculation of the Gibbs free energy of molecular association and dissociation. In the case of an association, the reactants have six translational and six rotational degrees of freedom in total, which transform in the product to three translational and three rotational degrees of freedom plus six new vibrational degrees of freedom. To avoid this error, Wertz[7] proposed an alternative semi-empirical mode of calculation of the translation-rotation entropy for a molecule in solution which consists of attributing to the dissolved molecule an energy equivalent to one-half the gas phase value (see also ref. [10]). In the present study, the contribution of the translation-rotation entropy to the reaction energy barrier is about 20 kcal mol$^{-1}$. According to Wertz's approach, it should be about half this value. In another approach, based on the analysis of data from the association of two weakly interacting molecules in aqueous solution, Yu et al.[9] concluded that the contribution of the translation-rotation entropy to the Gibbs free energy of association is less or equal to *9RT*. From this approach, our calculated reaction energy barrier should be corrected by at least -14.6 kcal mol$^{-1}$. By corroborating the two approaches, we conclude that a minimum correction of -10 kcal mol$^{-1}$ should be applied to the calculated energy barrier. The resulting predictive value for this barrier is 22 kcal mol$^{-1}$.

## 5. Cleavage of the S-C bond by direct insertion of a Hg atom

The alkyl group transfer between two thiolates could also occur indirectly through the formation of an intermediate product, in which mercury is inserted between the two atoms of the S-C bond. A first possibility is an insertion that occurs by an intramolecular rearrangement in a RS-Hg*-S*R* complex: Hg* is inserted between S* and R*, the R* group is transferred to SR, and the resulting R*SR group is eliminated. However, the scan of the potential energy surface of this system with respect to the Hg*-C* (from R*) distance (results not shown) invalidates this reaction pathway because it yields a significant increase of energy. Besides, a direct consequence of an insertion reaction should be the formation of an intermediate product in which the C* and S* atoms

are both bonded to Hg*. No energy minimum could be found for this molecular conformation.

A second possibility is an insertion reaction involving two RS-Hg-SR complexes. In this case, the Hg atom from one complex interacts with a thiolate group from the other complex. The corresponding insertion product was identified and its optimized structure shown in Figure S3. In a second reaction step, the $CH_3$ group bonded to Hg could be transferred to an adjacent S atom. The $CH_3$-S-$CH_3$ group thus formed could dissociate as in the case of the direct alkyl group transfer channel shown in Figure 1. The global reaction also is described by reaction (1) (main text). The difficulty with this model is that the Gibbs free energy of the insertion product is too high relative to that of the free reactants. A calculation performed at the ONIOM (CCSD(T)/MP2) level of theory using four explicit water molecules gives a positive difference of 49.0 kcal $mol^{-1}$. This is about 15 kcal $mol^{-1}$ higher than the energy barrier for the direct alkyl group transfer calculated under the same conditions (34.7 kcal $mol^{-1}$). Obviously, the transition state leading to the formation of this insertion product should be even higher in energy. We conclude that this pathway should be disregarded.

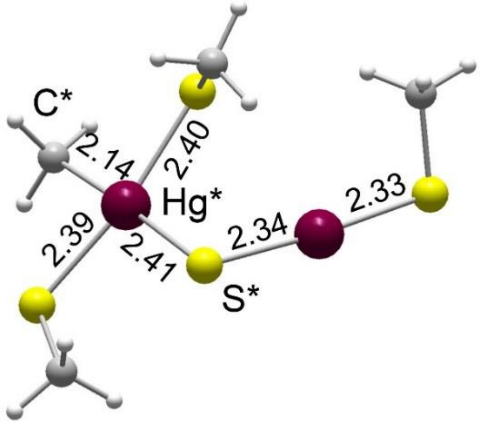


**Figure S3.** Formation of a Hg(II)-sulfide dimer by an insertion reaction; shown here is the intermediate product. Atoms directly involved in the reaction are marked with a star.

## 6. References

1 Marenich, A. V., Cramer, C. J. & Truhlar, D. G. Universal solvation model based on solute electron density and on a continuum model of the solvent defined by the bulk dielectric constant and atomic surface tensions. *J. Phys. Chem. B* **113**, 6378-6396 (2009).

2 Svensson, M. *et al.* ONIOM: A multilayered integrated MO+MM method for geometry optimizations and single point energy predictions. A test for Diels-Alder reactions and $Pt(P(t\text{-}Bu)_3)_2 + H_2$ oxidative addition. *J. Phys. Chem.* **100**, 19357-19363 (1996).

3 Dapprich, S., Komaromi, I., Byun, K. S., Morokuma, K. & Frisch, M. J. A new ONIOM implementation in Gaussian98. Part I. The calculation of energies, gradients, vibrational frequencies and electric field derivatives. *J. Mol. Str. - Theochem.* **461**, 1-21 (1999).

4 Manceau, A. *et al.* Formation of mercury sulfide from Hg(II)-thiolate complexes in natural organic matter. *Environ. Sci. Technol.* **49**, 9787−9796 (2015).

5 Reed, A. E., Curtiss, L. A. & Weinhold, F. Intermolecular interactions from a natural bond orbital, donor-acceptor viewpoint. *Chem. Rev.* **88**, 899-926 (1988).

6 Afaneh, A. T., Schreckenbach, G. & Wang, F. Y. Theoretical study of the formation of mercury ($Hg^{2+}$) complexes in solution using an explicit solvation shell in implicit solvent calculations. *J. Phys. Chem. B* **118**, 11271-11283 (2014).

7 Wertz, D. H. Relationship between the gas-phase entropies of molecules and their entropies of

solvation in water and 1-octanol. *J. Am. Chem. Soc.* **102**, 5316-5322 (1980).
8 Giesen, D. J., Cramer, C. J. & Truhlar, D. G. Entropic contributions to free-energies of solvation. *J. Phys. Chem.* **98**, 4141-4147 (1994).
9 Yu, Y. B., Privalov, P. L. & Hodges, R. S. Contribution of translational and rotational motions to molecular association in aqueous solution. *Biophys. J.* **81**, 1632-1642 (2001).
10 Lau, J. K. C. & Deubel, D. V. Hydrolysis of the anticancer drug cisplatin: Pitfalls in the interpretation of quantum chemical calculations. *J. Chem. Theor. Comput.* **2**, 103-106 (2006).

## 7. Gaussian 09 input files and optimized Cartesian coordinates

### *7.1. Optimization of the TS state with the pure cpcm solvation model*

```
%nprocshared=8
%mem=16GB
%chk=ts4c.chk
# opt(ts, readfc, maxstep=4, noeigentest) mp2/genecp scrf=cpcm

Title Card Required

0 1
 C    2.07720800    3.04341800    1.22928400
 S    1.82988400    2.39719100   -0.46252900
 C    4.30532800   -2.28022600    0.09052300
 S    2.48251000   -2.16510300    0.28647500
 Hg   2.15686200    0.10166200   -0.08937000
 H    1.40098300    2.53401800    1.93050600
 H    3.12043400    2.88627200    1.53349300
 H    4.60970700   -1.96452200   -0.91610400
 H    4.82062700   -1.67279600    0.84604100
 H    1.85427000    4.11962400    1.20617200
 H    4.56884200   -3.33862400    0.23341300
 C   -1.17498900   -2.77124600   -1.05433800
 S   -2.16838200   -2.40543800    0.45058300
 C   -0.43217700    2.34743200   -0.39315100
 S   -2.86573900    2.11796300   -0.26130700
 Hg  -2.57088800   -0.12888900    0.10125600
 H   -1.74005700   -2.51785200   -1.96203500
 H   -0.22806100   -2.21264000   -1.04001300
 H   -0.53722500    3.42896500   -0.45264500
 H   -0.40721700    1.86197400    0.57608000
 H   -0.95805100   -3.84966300   -1.05261900
 H   -0.48674300    1.75331700   -1.29862200

H C 0
aug-cc-pvdz
****
S  0
aug-cc-pvtz
****
Hg 0
sdd
****
Hg 0
F 1 1.00
    0.54500000    1.0000000
F   1   1.00
    1.5800000     1.0000000
****

HG     0
sdd
```

*7.2. Optimization of the IP state with the pure cpcm solvation model*

```
%nprocshared=8
%mem=16GB
%chk=ts4c_int1.chk
# opt mp2/genecp scrf=cpcm

Title Card Required

0 1
 C    0.17980900    2.84791100   -1.20214200
 S   -0.93138800    2.63841200    0.21830400
 C   -4.49933200   -1.16467600   -0.06311800
 S   -2.74973700   -1.66895500   -0.33068700
 Hg  -1.69904100    0.36940200   -0.02968400
 H    0.97657400    2.09196500   -1.15405400
 H   -0.43793300    2.73621300   -2.10346200
 H   -4.64315400   -0.77901300    0.95523700
 H   -4.80879500   -0.40723400   -0.79560400
 H    0.59586300    3.86366900   -1.14762700
 H   -5.11037000   -2.06976700   -0.19654700
 C    0.18292000   -2.60644700    1.50827200
 S    0.70851900   -2.45214800   -0.24441800
 C    0.25775500    2.57738700    1.58676900
 S    3.45318900    1.27025600    0.01295500
 Hg   2.04151800   -0.51607000   -0.12203100
 H    1.05558500   -2.66526300    2.17380100
 H   -0.45389000   -1.75866400    1.79678400
 H    0.63005000    3.59990500    1.74182900
 H    1.07893400    1.89574200    1.32287200
 H   -0.40267000   -3.53393300    1.59542300
 H   -0.29461500    2.23368800    2.47142800

H C 0
aug-cc-pvdz
****
S  0
aug-cc-pvtz
****
Hg 0
sdd
****
Hg 0
F 1 1.00
    0.54500000    1.0000000
F   1   1.00
    1.5800000     1.0000000
****

HG     0
sdd
```

*7.3. Optimization of the PC state with the pure cpcm solvation model*

```
%nprocshared=8
%mem=16GB
%chk=pc4b.chk
# opt mp2/genecp scrf=cpcm

Title Card Required

0 1
 C   -1.23334900   -1.06068600    2.58002000
 S   -2.72630800   -1.39188500    1.60891200
 C   -0.60489600    3.43738400   -0.00182500
```

```
 S   -1.88522600    2.21149500    0.49138600
 Hg  -1.09706500    0.36485700   -0.71071500
 H   -0.35698600   -1.01946900    1.91490400
 H   -1.36739200   -0.08158700    3.06104700
 H    0.40385800    3.04474100    0.19312600
 H   -0.69701700    3.69107700   -1.06697800
 H   -1.09311500   -1.83885300    3.34556500
 H   -0.76577400    4.34230100    0.60261100
 C    1.79532400    1.16188300    2.50166200
 S    3.04327500    0.52267000    1.31048200
 C   -2.20527000   -2.95319600    0.84822300
 S    0.09343400   -1.31165900   -1.83681900
 Hg   1.62995300   -0.44296200   -0.27817700
 H    0.99319300    1.70360100    1.98087000
 H    1.35999500    0.33915400    3.08530400
 H   -2.00372300   -3.70807000    1.62332200
 H   -1.30941700   -2.77758400    0.23216600
 H    2.32089700    1.84897700    3.18110500
 H   -3.02923200   -3.29813900    0.20646700

H C 0
aug-cc-pvdz
****
S  0
aug-cc-pvtz
****
Hg 0
sdd
****
Hg 0
F 1 1.00
    0.54500000    1.0000000
F   1   1.00
    1.5800000     1.0000000
****

HG     0
sdd
```

*7.4. Optimization of the TS state with the cpcm solvation model and 4 explicit water molecules*

```
%nprocshared=12
%mem=44GB
%chk=ts4c_4w.chk
# opt(ts, modredundant, noeigentest, maxstep=4) mp2/genecp scrf=cpcm

Title Card Required

0 1
 C    2.12227700   -3.16317800   -0.87031500
 S    2.18309800   -2.28906200    0.73496000
 C    2.67192900    3.02234400    0.93857900
 S    2.65507700    2.15417900   -0.68201500
 Hg   2.44573200   -0.05029500    0.01917100
 H    1.36250400   -2.70540400   -1.51940500
 H    3.10870800   -3.12149300   -1.35003300
 H    1.75225900    2.81650600    1.50201000
 H    3.54629500    2.72069600    1.53044700
 H    1.85565500   -4.20849900   -0.65709100
 H    2.73664100    4.09761200    0.71674400
 C   -1.03310600    3.12885600   -0.25966400
 S   -2.12828200    2.15371900   -1.36719000
 C   -0.09046400   -2.12341100    0.90551100
 S   -2.50021500   -1.88538700    0.89778000
 Hg  -2.39404900    0.16227100   -0.15954200
```

```
 H   -1.47204400    3.22018600    0.74278100
 H   -0.03595200    2.66986400   -0.20287600
 H   -0.11230600   -1.71484000   -0.09731000
 H   -0.03848300   -1.45462000    1.75945700
 H   -0.93630400    4.12930000   -0.70745000
 H   -0.21494200   -3.19354200    1.05799600
 O   -1.48974700   -2.70833800   -2.04801900
 H   -2.23226600   -2.83125100   -2.65738700
 H   -1.92426400   -2.53554900   -1.17869900
 O   -2.37443700    0.12635500    3.40942100
 H   -3.10425600    0.75205200    3.28960400
 H   -2.51231100   -0.52976600    2.68412900
 O    0.09846400    1.05981100    2.43036400
 H    0.00706100    1.00098900    1.46844500
 H   -0.78585000    0.78844500    2.75928400
 O    0.07199400   -0.40585400   -2.63415500
 H   -0.42194800    0.38601100   -2.35797000
 H   -0.54169900   -1.14343700   -2.43504000

B 2 14 D
B 14 15 D

H C O 0
aug-cc-pvdz
****
S  0
aug-cc-pvtz
****
Hg 0
sdd
****
Hg 0
F 1 1.00
    0.54500000    1.0000000
F   1   1.00
    1.5800000     1.0000000
****

HG     0
sdd
```

*7.5. Optimization of the IP state with the cpcm solvation model and 4 explicit water molecules*

```
%nprocshared=12
%mem=24GB
%chk=ts4c_int1_4w_opt.chk
# opt mp2/genecp scrf=cpcm

Title Card Required

0 1
 C   -0.54970800    2.53311000   -1.35447800
 S   -1.52715100    2.09573700    0.11181500
 C   -3.99131900   -2.50856400    0.07000900
 S   -2.17924900   -2.55258800   -0.24812000
 Hg  -1.68580600   -0.30219900   -0.04414600
 H    0.41708900    2.01131500   -1.31495500
 H   -1.14284600    2.23245500   -2.22869300
 H   -4.20110100   -2.13606600    1.08182900
 H   -4.50780000   -1.88434600   -0.67136100
 H   -0.41040100    3.62325600   -1.34399400
 H   -4.34987600   -3.54514500   -0.01512700
 C    0.94779900   -2.63144100    1.50574100
 S    1.36476600   -2.41090600   -0.26894200
 C   -0.32418400    2.39459800    1.43608500
```

```
 S    3.05457300    1.90160000   -0.21840000
 Hg   2.15207700   -0.19277900   -0.25059300
 H    1.82452900   -2.43803200    2.13969900
 H    0.12120800   -1.96719600    1.79389900
 H   -0.22595300    3.48361500    1.54843000
 H    0.63794400    1.93934700    1.16098400
 H    0.62598000   -3.67496500    1.64060400
 H   -0.74268900    1.95247600    2.34997400
 O    5.65887800    0.06200000   -0.93801100
 H    6.39169600    0.68462400   -1.04326700
 H    4.89142600    0.64189300   -0.71705600
 O   -5.27396300    0.83433900    0.67731400
 H   -5.01705100    1.60706300    0.13496700
 H   -4.64593800    0.14779200    0.41712300
 O   -4.53077100    3.09331400   -0.87839500
 H   -3.78946800    3.51272700   -0.41679200
 H   -5.19079200    3.79692400   -0.95933100
 O    2.62205200    1.43511700    2.97569800
 H    2.78869800    1.55963400    2.01042300
 H    3.51157100    1.38499300    3.35306500

H C O 0
aug-cc-pvdz
****
S  0
aug-cc-pvtz
****
Hg 0
sdd
****
Hg 0
F 1 1.00
    0.54500000    1.0000000
F   1   1.00
    1.5800000     1.0000000
****

HG     0
sdd
```

*7.6. Optimization of the PC state with the cpcm solvation model and 4 explicit water molecules*

```
%nprocshared=8
%mem=16GB
%chk=pc4b_opt.chk
# opt mp2/genecp scrf=cpcm

Title Card Required

0 1
 C    2.10184000    1.21304400    1.58397700
 S    3.10356800    0.74845600    0.14724200
 C   -0.68569800   -0.79815900    3.20597100
 S    0.27871900   -2.13523000    2.38931700
 Hg   0.02417000   -1.46280900    0.16772500
 H    1.03830000    1.14513900    1.31699300
 H    2.32477300    0.49869100    2.38889000
 H   -0.21390700    0.18071800    3.04054800
 H   -1.71795300   -0.77588600    2.83000500
 H    2.34839100    2.23680000    1.90297400
 H   -0.69236100   -1.01904900    4.28340800
 C   -0.92209400    3.35222900    1.56762400
 S   -2.41006000    2.54661000    0.85423800
 C    2.41338600    1.91059400   -1.06014300
 S   -0.47783600   -0.64527600   -1.99319400
```

```
 Hg  -1.44112000    0.95348200   -0.55063200
 H   -0.42287900    2.68639700    2.28458900
 H   -0.21607000    3.64559400    0.77904600
 H    2.52084000    2.94058500   -0.68853900
 H    1.35604200    1.66853500   -1.23714200
 H   -1.27072400    4.25136600    2.09654200
 H    2.97726000    1.77820300   -1.99240800
 O    2.53920300   -0.45920400   -3.35009100
 H    2.28515200   -0.08810400   -4.20675900
 H    1.70001800   -0.45701800   -2.84837700
 O   -3.30074800   -2.13744000   -0.91122000
 H   -3.02016300   -2.18236500    0.01492200
 H   -2.53298300   -1.71004000   -1.34025400
 O    3.26752100   -2.61309400   -0.37888600
 H    4.20918500   -2.57350800   -0.59826300
 H    3.01976000   -1.67594800   -0.28520900
 O    5.54412700    2.94642000    0.56801800
 H    4.85132900    2.26029500    0.50297000
 H    5.05628900    3.74310400    0.81981700

H C O 0
aug-cc-pvdz
****
S  0
aug-cc-pvtz
****
Hg 0
sdd
****
Hg 0
F 1 1.00
    0.54500000    1.0000000
F   1   1.00
    1.5800000     1.0000000
****

HG     0
sdd
```

### 7.7. *Optimization of the TS state with the cpcm solvation model and 7 explicit water molecules*

```
%nprocshared=12
%mem=24GB
%chk=ts4c_7w.chk
# opt=(ts,modredundant,maxstep=1,noeigentest) mp2/genecp scrf=cpcm

Title Card Required

0 1
 C   -1.66306000    2.05262400   -2.63993700
 S   -1.83842400    2.05674800   -0.81874400
 C   -2.71009500   -2.52847600    1.79178600
 S   -2.52447200   -2.50756700   -0.03757700
 Hg  -2.17387400   -0.24074500   -0.41054200
 H   -0.87467400    1.34509000   -2.93190300
 H   -2.61883800    1.76512300   -3.09724800
 H   -1.82121800   -2.11041700    2.28258300
 H   -3.59994700   -1.96205400    2.09705400
 H   -1.39427600    3.07449800   -2.94306200
 H   -2.82906400   -3.58228000    2.08319700
 C    1.11833700   -3.12077100    1.17390300
 S    2.16288000   -2.71095200   -0.28215600
 C    0.40489000    2.05312400   -0.37425400
 S    2.78281400    1.87982600    0.04132800
 Hg   2.54062000   -0.42704700    0.03111800
```

```
H    1.56740800   -2.73157900    2.09734900
H    0.10381000   -2.72040800    1.03790200
H    0.50546700    1.23210700   -1.07325100
H    0.22220500    1.85104600    0.67648200
H    1.06177500   -4.21771000    1.23146600
H    0.52795900    3.07820700   -0.71751700
O    2.14069300    1.21655000   -3.05913400
H    2.94784400    1.01456100   -3.55445000
H    2.46919100    1.46698600   -2.16433300
O    2.07110600    1.33909000    3.14095700
H    2.76129300    0.79314800    3.54560500
H    2.40975700    1.51083500    2.23013800
O   -2.65630600    1.40304100    2.81800700
H   -1.88406300    0.87232300    2.54166300
H   -3.30424400    1.33596800    2.09380600
O   -0.23361700   -0.04944900    2.47221900
H    0.00459700   -0.49042300    1.64431600
H    0.59544900    0.41036400    2.73937700
O   -4.79391000    1.58782400    0.81016300
H   -4.20414500    1.92503000    0.11291100
H   -5.01903600    0.70021400    0.49134000
O    0.35729400   -0.97731200   -2.72629800
H    0.71447700   -1.55186100   -2.02463300
H    1.04485400   -0.28668400   -2.82717000
O    1.28611900    4.46203200    1.46261100
H    1.86041000    4.93225200    2.08305900
H    1.81705600    3.68691400    1.19648300

1 2 1.0 6 1.0 7 1.0 10 1.0
2 5 1.0
3 4 1.0 8 1.0 9 1.0 11 1.0
4 5 1.0
5
6
7
8
9
10
11
12 13 1.0 17 1.0 18 1.0 21 1.0
13 16 1.0
14 19 1.0 20 1.0 22 1.0
15 16 1.0
16
17
18
19
20
21
22
23 24 1.0 25 1.0
24
25
26 27 1.0 28 1.0
27
28
29 30 1.0 31 1.0
30
31
32 33 1.0 34 1.0
33
34
35 36 1.0 37 1.0
36
37
```

```
 38 39 1.0 40 1.0
 39
 40
 41 42 1.0 43 1.0
 42
 43

B 2 14 D
B 14 15 D
B 15 25 B
B 15 28 B
B 15 43 B
B 2 36 B
B 2 30 B

H C O 0
aug-cc-pvdz
****
S  0
aug-cc-pvtz
****
Hg 0
sdd
****
Hg 0
F 1 1.00
    0.54500000    1.0000000
F   1   1.00
    1.5800000     1.0000000
****

HG     0
sdd
```
